# *OsARF4* regulates leaf inclination *via* auxin and brassinosteroid pathways in rice

**DOI:** 10.3389/fpls.2022.979033

**Published:** 2022-09-30

**Authors:** Jiyue Qiao, Yanjun Zhang, ShaqiLa Han, Senqiu Chang, Zhenyu Gao, Yanhua Qi, Qian Qian

**Affiliations:** ^1^State Key Laboratory of Rice Biology, China National Rice Research Institute, Chinese Academy of Agricultural Sciences, Hangzhou, China; ^2^Hainan Yazhou Bay Seed Laboratory, Sanya, China; ^3^Key Laboratory of Herbage and Endemic Crop Biology of Ministry of Education, Inner Mongolia Key Laboratory of Herbage and Endemic Crop Biotechnology, School of Life Sciences, Inner Mongolia University, Hohhot, China

**Keywords:** leaf inclination, auxin, *OsARF4*, rice, brassinosteroid

## Abstract

Leaf inclination is a vital agronomic trait and is important for plant architecture that affects photosynthetic efficiency and grain yield. To understand the molecular mechanisms underlying regulation of leaf inclination, we constructed an *auxin response factor (arf)* rice mutant—*osarf4*—showing increased leaf inclination using CRISPR/Cas9 gene editing technology. *OsARF4* encodes a nuclear protein that is expressed in the lamina joint (LJ) at different developmental stages in rice. Histological analysis indicated that an increase in cell differentiation on the adaxial side resulted in increased leaf inclination in the *osarf4* mutants; however, *OsARF4*-overexpressing lines showed a decrease in leaf inclination, resulting in erect leaves. Additionally, a decrease in the content and distribution of indole-3-acetic acid (IAA) in *osarf4* mutant led to a greater leaf inclination, whereas the *OsARF4*-overexpressing lines showed the opposite phenotype with increased IAA content. RNA-sequencing analysis revealed that the expression of genes related to brassinosteroid (BR) biosynthesis and response was different in the mutants and overexpressing lines, suggesting that *OsARF4* participates in the BR signaling pathway. Moreover, BR sensitivity assay revealed that *OsARF4*-overexpressing lines were more sensitive to exogenous BR treatment than the mutants. In conclusion, OsARF4, a transcription factor in auxin signaling, participates in leaf inclination regulation and links auxin and BR signaling pathways. Our results provide a novel insight into l leaf inclination regulation, and have significant implications for improving rice architecture and grain yield.

## Introduction

Plant architecture is governed by a set of complex agronomic traits that determine grain yield and is a pivotal target of artificial selection for rice domestication. Rice tillering pattern, plant height, and leaf inclination are important factors influencing the plant architecture (Van Camp, 2005). Leaf inclination (also called leaf angle), which is the angle between the leaf blade and the culm, is determined by the lamina joint (LJ). LJ, which is composed of a collar, ligules, and auricles, is a unique tissue that connects the leaf blade to leaf sheath in rice, and significantly contributes to the horizontal bending of the blade from the main axis. Division and expansion of the parenchyma and sclerenchyma cells in LJ affect its development, and leaf inclination is influenced by unequal cell division and elongation between the adaxial and abaxial sides of the LJ. Lack of cell expansion and longitudinal elongation of adaxial cells in the LJ results in erect leaves. In contrast, leaf inclination is enhanced by an increase in the expansion of cells on the adaxial surface (Duan et al., 2006). In rice, erect leaves have a higher leaf area index, which can increase the photosynthetic carbon assimilation rate by increasing light capture ability and nitrogen use efficiency to enhance total biomass and grain yield (Sakamoto et al., 2006). Therefore, revealing the molecular mechanism of genes involved in regulation of leaf inclination in rice is urgently required.

Phytohormones play an important role in regulating leaf inclination by changing the cytological structure of LJ. Brassinosteroids (BRs), plant-specific steroid hormones, have a significant effect on the growth and development of the LJ. In rice, studies have shown that BR promotes division and elongation of parenchyma cells at the adaxial side of the LJ and proliferation of sclerenchyma cells at the abaxial side; this unbalanced development between the adaxial and abaxial cells of the LJ leads to leaf bending (Tanabe et al., 2005; Zhang L. Y. et al., 2009; Sun et al., 2015). Several studies have indicated that decreased BR biosynthesis and signal transduction could decrease leaf inclination, whereas excess BR results in increased leaf inclination (Zhang et al., 2014). The mutation of several cytochrome P450 genes involved in BR biosynthesis, such as *OsD2*, *OsD11,* and *OsDWARF* (Hong et al., 2003; Sakamoto et al., 2006; Li et al., 2013), causes shortening of parenchyma cells in the LJ, contributing to reduced leaf inclination and formation of erect leaves. Defective BR signaling genes—*BAK1* and *BZR1*—also result in decreased leaf inclination (Bai et al., 2007; Li et al., 2009). *RLA1/SMOS1 (REDUCED LEAF ANGLE1/ SMALL ORGAN SIZE1)* acts as an integrator, functioning with OsBZR1 and GSK2, and plays an important role in BR signal transduction and leaf inclination regulation in rice (Qiao et al., 2017). Furthermore, *ILI1* (*INCLINATION1*) and *IBH1* (*ILI1 binding bHLH*) antagonistically regulate the elongation of parenchyma cells in the LJ by interacting with the transcription factor OsBZR1 involved in BR signaling (Zhang L. Y. et al., 2009). In addition, rice U-type cyclin CYCU4; 1 regulates proliferation of sclerenchyma cells on the abaxial side of LJ in the BR-regulated pathway, which plays an important role in promoting the erectness of leaves (Sun et al., 2015). These results suggest that leaf inclination is closely related to BR biosynthesis and signal transduction in rice.

In addition to BR, auxin has been shown to play a negative role in controlling leaf inclination. Studies have shown that reducing auxin levels or signal transduction increases leaf inclination by promoting the elongation of parenchyma cells on the adaxial surface of the LJ (Du et al., 2012; Zhao et al., 2013; Zhang et al., 2015). Furthermore, loss of *FIB* (*Fish Bone*) function could reduce the endogenous indole acetic acid (IAA) content and polar auxin transport activity, leading to increased bending of the LJ (Yoshikawa et al., 2014). Auxin early response genes, such as *AUX/IAA* and *GH3* family genes, the auxin receptor TIR1 (TRANSPORT INHIBITOR RESPONSE 1), and auxin response factor (ARF), have been reported to be associated with regulation of leaf inclination. The auxin response factor *OsARF1* can inhibit the expression of *OsGH3s*, which encode IAA amino acid synthases by interacting with the repressor *OsIAA1* (*AUXIN/INDOLE3-ACETIC ACID 1*) in the process of leaf inclination regulation (Wang et al., 2007). Additionally, overexpression of *OsIAA1* results in an increased leaf angle (Song et al., 2009). Recent studies have shown that *OsIAA6* regulates leaf inclination by inhibiting auxin signaling through interacting with *OsARF1* in rice (Xing et al., 2022). The decrease in IAA content promotes elongation of parenchyma cells on the adaxial surface of the LJ, which is consistent with the result that the increase in *GH3* expression levels increases leaf inclination. The increase in *GH3–5* expression levels and the decrease in free IAA content also lead to increased leaf inclination in *OsARF19*-overexpressing rice plants (Zhang et al., 2015). *LC1* (*LEAF INCLINATION 1*) encodes OsGH3-1 (an IAA amino synthetase) and promotes cell elongation at the LJ by decreasing the auxin content in rice (Zhao et al., 2013). *LC3* (*LEAF INCLINATION 3*) interacts with *LIP1* (*LC3-INTERACTING PROTEIN 1*) to synergistically suppress auxin signaling, thus controlling leaf inclination (Chen et al., 2018). *LPA1* (*LOOSE PLANT ARCHITECTURE 1*) influences the polar transport of auxin by regulating the expression of auxin transporter *OsPIN1a* and influences leaf angle regulation (Sun et al., 2019). Inhibition of the expression of auxin receptor OsTIR1 also leads to increased leaf inclination (Bian et al., 2012). In addition, *OsARF6* and *OsARF17* synergistically control flag leaf angle in response to auxin (Huang et al., 2021).

Both auxin and BR certainly play important roles in regulation of leaf inclination, which involves diverse factors and intricated regulatory networks. In this regard, we identified a novel gene *OsARF4* (*AUXIN RESPONSE FACTOR 4*), mutants of which showed increased leaf inclination, whereas overexpressing lines had erect leaves. Histochemical analysis and scanning electron microscopy (SEM) results showed that the increase in leaf inclination was caused by cell differentiation on the adaxial surface. Moreover, *OsARF4* was found to be involved in both auxin and BR signaling in regulation of leaf inclination. These results provide a new potential target for ideal architecture breeding and improving rice yield.

## Materials and methods

### Plant materials and growth conditions

Wild type variety of rice—Dongjin (*Oryza sativa* subsp. *japonica*, WT/DJ)—was used as a control in this study. All seeds were soaked in water in the dark for 3 days at 37°C, and then cultured in a nutrient solution (pH 5.4) and grown in the greenhouse under a 12 h light (30°C) and 12 h dark (24°C) cycle. Thereafter, rice plants were grown in an experimental paddy field under natural conditions of the China National Rice Research Institute in Hangzhou or Hainan Yazhou Bay Seed Laboratory in Sanya, China.

### Construction and transformation of binary vectors

The *osarf4* mutants were obtained using CRISPR/Cas9 gene editing technology as described by Xie (Xie et al., 2015), and the selected target sites of *osarf4* mutants were AGGAGGCATCTCCTTCAGAG and AGTTCCAAAAGGCTTGTTGC. The full-length open reading frame (ORF) of *OsARF4* was amplified from WT/DJ cDNA and then cloned into *pCAMBIA1300-sGFP* binary vector to construct the overexpression vector *35S:OsARF4-GFP*. *OsARF4* promoter was amplified from WT/DJ genomic DNA and then cloned into the *pCAMBIA1301-GUS* vector to generate *proOsARF4:GUS* vector. *Agrobacterium* strain EHA105 was used for transformation of the vector into WT/DJ. All primers used are shown in Supplementary Table 1.

### RNA extraction and quantitative real-time PCR (qRT-PCR)

Total RNA was extracted using TaKaRa MiniBEST Plant RNA Extraction Kit (TaKaRA, Kusatsu, Japan) and then reverse transcribed to obtain cDNA using FastKing RT Kit (with gDNase; Tiangen, Beijing, China) according to the manufacturer’s instructions. Quantitative real-time PCR (qRT-PCR) was performed using the 2*Easy Star Green Fast Mixture (Easy-Do, Zhejiang, China) and a CFX Connect™ Real-time System (Bio-Rad, Hercules, CA, United States). *OsACTIN* (*LOC_Os03g50885*) was used as an internal control. Three biological replicates were performed for each experiment. All primers used for qRT-PCR are shown in Supplementary Table 2.

### Subcellular localization of *OsARF4*

High-quality plasmids of *35S:OsARF4-GFP* and the nuclear localization marker NSL-*mCherry* were transiently coexpressed in rice protoplasts using polyethylene glycol as previously described (Qiao et al., 2021). After overnight incubation in the dark at 28°C, fluorescence was observed using a two-photon confocal microscope (Zeiss LSM 710; Carl Zeiss, Oberkochen, Germany).

### *β*-Galactosidase (GUS) staining and activity analysis

The auxin reporter *DR5:GUS* was genetically transformed into WT/DJ, *osarf4* mutants, and *OsARF4*-overexpressing lines. The LJs were treated with the GUS staining solution (10 mM sodium phosphate buffer, pH 7.0; 10 mM Na_2_EDTA; 1 mM K_3_[Fe(CN)_6_]; 1 mM K_3_[Fe(CN)_6_]; 0.5% Triton X-100; 20% methyl alcohol, and 0.5 mg/ml X-Gluc) for 30 min at 37°C. Then, chlorophyll was removed by soaking the leaves in 70% ethanol. Images were collected using a stereoscope Nikon SMZ 25 (Nikon Corporation, Tokyo, Japan). The positive *pro:OsARF4-GUS* transgenic lines were tested by GUS staining, and roots (3-day-old of seedlings), coleoptile (3-day-old seedlings), and 2–10 week old of seedings of *pro:OsARF4-GUS* were used for expression pattern analysis. GUS activities in the LJs of WT/DJ, *osarf4* mutants, and *OsARF4*-overexpressing lines were analyzed using the Plant GUS Elisa Kit^1^ according to the manufacturer’s instructions.

### Quantification of IAA contents

At 10 days after heading, the LJs of WT/DJ, *osarf4* mutants, and *OsARF4*-overexpressing lines were ground into a powder using liquid nitrogen. IAA was extracted and its content was measured as previously described (Ljung et al., 2005; Shao et al., 2019; Qiao et al., 2021). Agilent 1,100 high-performance liquid chromatography was performed using a C18 reverse-phase column (250 mm × 4.6 mm, 5 m).

### Scanning electron microscopy

Scanning electron microscopy was performed as previously described (Zhao et al., 2010; Wang et al., 2019). Briefly, at 10 days after heading, approximately 1 cm of LJs was excised from the flag leaves of WT/DJ, *osarf4* mutants, and *OsARF4*-overexpressing lines. Thereafter, they were dehydrated in graded ethanol series (50–100%), dried in a critical point dryer for 2 h, and subjected to gold sputtering for 1 min. Samples were observed using an S-3000 N scanning electron microscope (Hitachi, Tokyo, Japan).

### Leaf inclination measurement and cytological analysis

Leaf inclinations between the sheaths and leaves of WT/DJ, *osarf4* mutants, and *OsARF4*-overexpressing lines were photographed and measured at 10 days after heading. For paraffin sectioning, the LJs from flag leaves of WT/DJ, *osarf4* mutants, and *OsARF4*-overexpressing lines were taken and fixed using FAA solution (37% formaldehyde, acetic acid, 70% alcohol, 5:5:90, v:v:v), followed by dehydration in a graded ethanol series and embedding in Paraplast Plus (Sigma, United States). Microtome sections (8 μm) were cut to obtain transverse and longitudinal sections using a rotary microtome (RM2245, Leica Microsystems, Hamburg, Germany) and stained with toluidine blue. Sections were observed and photographed using a stereoscope Nikon SMZ 25 (Nikon Corporation, Tokyo, Japan).

### RNA sequencing analysis

At 10 days after heading, total RNA was extracted from LJs of flag leaves of WT/DJ, *osarf4-1* mutant, and *OE-OsARF4-1* overexpression line, and sequenced in BGI with three biological replicates (Shenzhen, China). The analytical methods and software analysis were performed according to previous studies (Zhang et al., 2020). Gene expression levels were expressed as transcript fragments per kilobase (FPKM) per million reads (Florea et al., 2013). Differentially expressed genes (DEGs) were detected using the DESeq 2 package testing at |log2FC| > = 2 and Q value <= 0.05. Additional detailed information is provided on the BGI official website.^2^ The RNA sequencing data were deposited in the NCBI Sequence Read Archive (SRA) with accession SRR20748275, SRR20748276, SRR20748277, SRR20748278, SRR20748279, SRR20748280, SRR20748281, SRR20748282, and SRR20748283.

### BR sensitivity tests

Primary root (PR) inhibition analysis was performed as previously mentioned (Zhang et al., 2015). Seeds of WT/DJ, *osarf4* mutants, and *OsARF4*-overexpressing lines were germinated at 37°C and then grown in a normal culture solution supplemented with 0, 0.01, 0.1, 1, or 10 μm 24-epibrassinolide (24-eBL) for 7 days. Then, the PR length was measured. The coleoptile elongation test was performed as previously described (Duan et al., 2006). Seeds of WT/DJ, *osarf4* mutants, and *OsARF4*-overexpressing lines were germinated and grown in a normal culture solution containing 0, 0.01, 0.1, 1, or 10 μm 24-eBL for 7 days. Then, a camera (EOS 6D Mark II, Canon, Japan) was used to obtain a photograph to measure the coleoptile length. After 7 days of growth in a normal culture solution, WT/DJ, *osarf4*, and *OsARF4*-overexpressing plants were incubated with 24-eBL at different concentrations for 3 days. Thereafter, the leaf inclinations were measured as previously described (Zhang et al., 2015).

## Results

### *OsARF4* is expressed in lamina joint and encodes a nuclear protein

**Figure 1:**
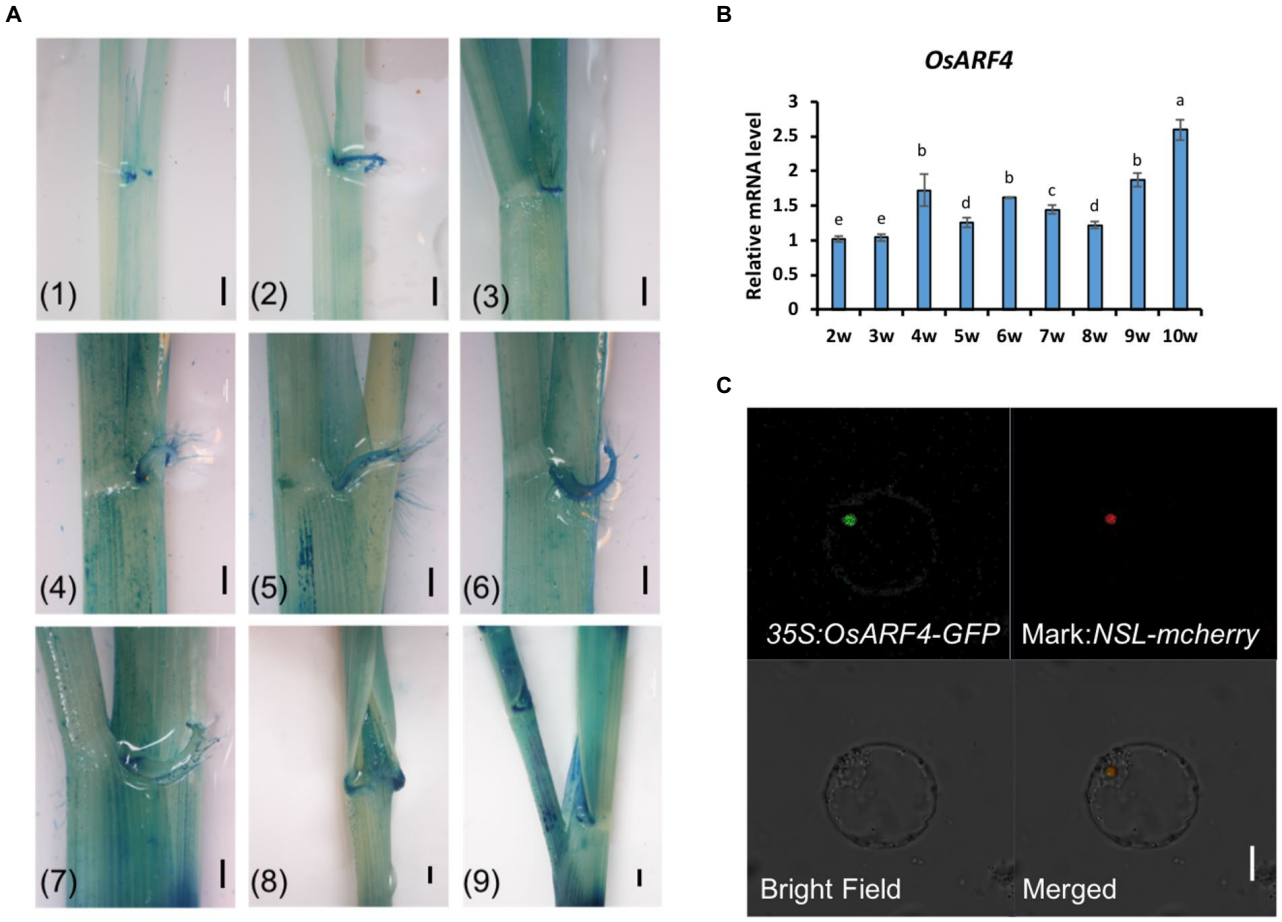
Expression patterns of the *OsARF4* gene and subcellular localization of OsARF4. **(A)** The expression patterns of the *OsARF4* gene. (1)–(9) show GUS staining of 2, 3, 4, 5, 6, 7, 8, 9, and 10-week-old seedlings, respectively. Scale bar = 1 mm. **(B)** qRT-PCR analysis of *OsARF4* expression in WT/DJ rice. Three separate biological replicates were used in this experiment. Error bars represent SD (*n* = 3). Lowercase letters represent significance according to Duncan’s multiple range test (α = 0.05). **(C)** Subcellular localization of OsARF4 in rice. *35S:OsARF4-GFP* and the nuclear localization marker NLS-*mCherry* were transiently coexpressed in rice protoplasts. Scale bar = 20 mm.

Our previous study has found that the member of the *OsARF* family, *OsARF19*, controls rice leaf inclination positively (Zhang et al., 2015). Although *OsARF4* and *OsARF19* belong to the *OsARF* family, they exist in different clades. *OsARF4* belongs to Class I subfamily, while *OsARF19* is from Class II (Wang et al., 2007). OsARF4 is a putative transcriptional repressor with RD (repression domain) in its middle region (MR), while OsARF19 is a putative transcriptional activator with activation domain (AD; Shen et al., 2010). Furthermore, *OsARF4* has a negative function in regulating grain size and grain weight in rice (Hu et al., 2018). In that case, whether *OsARF4* also takes part in leaf inclination regulation? In order to make this question clearly, the related tests were performed. To determine the functional location of *OsARF4*, its expression pattern was first investigated. The promoter of *OsARF4* was fused with the *GUS* gene and this construct was then transformed into WT/DJ calluses to obtain the *pro:OsARF4-GUS* transgenic plants. *Pro:OsARF4-GUS* transgenic plants were observed at different stages during 2–10 weeks using histochemical staining. The results showed that *OsARF4* was expressed in LJ at different development stages, especially at the mature stage (Figure 1A). qRT-PCR further confirmed that *OsARF4* was expressed at those stages in LJ (Figure 1B), consistent with the results of GUS staining, implying that *OsARF4* may function in LJ in rice.

To explore the subcellular localization of OsARF4, the CDS of *OsARF4* was fused with GFP and driven by the CaMV35S promoter. Transient co-expression of *35S:OsARF4-GFP* and the nuclear marker NLS-*mcherry* in rice protoplasts showed that OsARF4 was localized in the nucleus (Figure 1C), Furthermore, OsARF4 was also localized in the nuclei of root cells in *35S:OsARF4-GFP* transgenic lines (Supplementary Figure 1). These results indicate that *OsARF4* encodes a nuclear protein.

### *OsARF4* negatively affects leaf inclination

**Figure 2:**
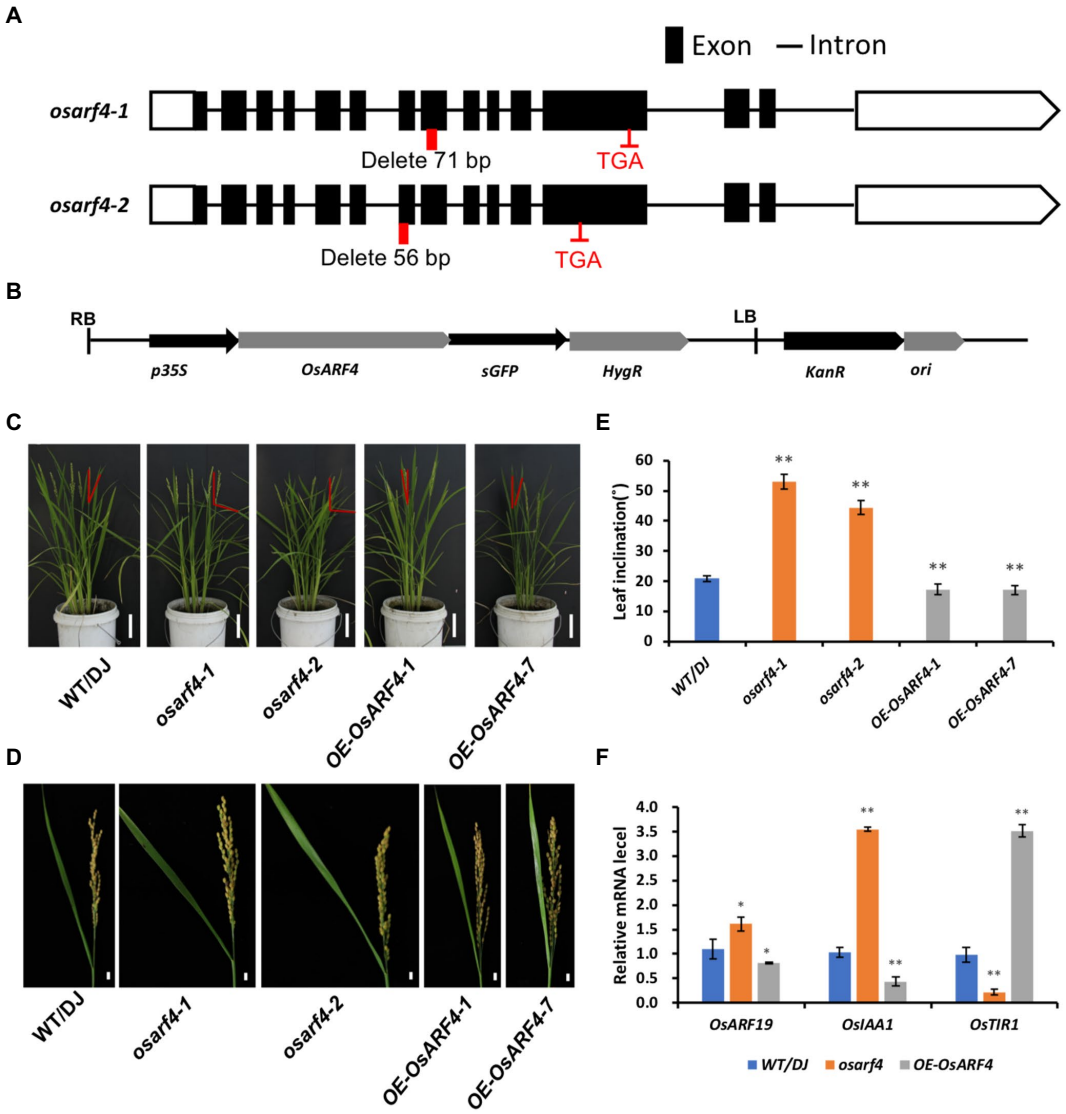
Phenotypic characterization of *OsARF4*. **(A)** Construction of two *osarf4* mutants using CRISPR/Cas9 technology. Black lines, black boxes, and red boxes indicate introns, exons, and editing sites, respectively. TGA is a stop codon. **(B)** Map of the *OsARF4-*overexpressing vector. **(C)** Phenotypes of 3-month-old seedlings of WT/DJ, *osarf4* mutants (*osarf4-1* and *osarf4-2*), and *OsARF4-*overexpressing lines (*OE-OsARF4-1* and *OE-OsARF4-7*). Bars = 10 cm. **(D)** Flag leaf angles of WT/DJ, *osarf4* mutants, and *OsARF4-*overexpressing seedlings at mature stages. Bars = 3 cm. **(E)** Quantification of flag leaf angles at mature stages. Ten biological replicates were taken for each experiment. ** indicates a significant difference at *p* < 0.01. **(F)** Relative expression levels of leaf inclination-related genes in WT/DJ, *osarf4* mutants, and *OsARF4*-overexpressing lines. Three individual biological replicates were taken for each experiment. *p < 0.05 and **p < 0.01 indicate significant differences compared with WT/DJ using Student’s *t*-test.

Studies have shown that *OsARF* gene family members *OsARF19*, *OsARF6,* and *OsARF1* were involved in regulating leaf inclination in rice (Zhang et al., 2015; Huang et al., 2021; Xing et al., 2022). To explore whether *OsARF4* functions to control leaf inclination, two *osarf4* mutants were constructed using CRISPR/Cas9 gene editing technology (Xie et al., 2015), and two *OsARF4*-overexpressing lines were obtained (Figures 2A,B). Compared with WT/DJ, *osarf4-1* showed a 71-bp deletion in the eighth exon in the ORF (1834–1904 bp) of *OsARF4*, which caused premature termination of protein translation, resulting in the formation of 627 amino acid protein instead of the 808 amino acid protein in *osarf4-1*. *osarf4-2* showed a 56-bp deletion in the seventh exon in the ORF (1631–1,684 bp), which caused premature termination of protein translation resulting in the formation of a truncated protein with 564 amino acids instead of 808 amino acids. The protein structure in both mutants was changed (Figure 2A). The expression of *OsARF4* was upregulated four and nine folds in *OsARF4-1* and *OsARF4-7*-overexpressing lines, respectively, compared with that in WT/DJ (Supplementary Figure 2). Phenotypic observation showed that at the heading stage, leaf angles of *osarf4* mutants (*osarf4-1* and *osarf4-2*) were much larger than that of WT/DJ, and the *OsARF4*-overexpressing lines (*OE-OsARF4-1* and *OE-OsARF4-7*) showed a greater number of erect leaves than inclined leaves (Figure 2C). This was more evident in the flag leaves (reaching to ~85° in *osarf4* mutants and ~ 18° in *OsARF4*-overexpressing lines compared with 28° in WT/DJ; Figures 2D,E).

To further verify the function of *OsARF4* in controlling leaf inclination, we analyzed the expression of genes related to regulation of leaf inclination in the LJs of WT/DJ, *osarf4* mutants, and *OsARF4*-overexpressing lines (Figure 2F). The transcriptional abundances of the genes *OsARF19* and *OsIAA1* were induced in the *osarf4* mutants, whereas they were reduced in *OsARF4*-overexpressing lines compared with those in WT/DJ. In addition, *OsTIR1* showed a opposite trend compared with *OsIAA1*. These results are consistent with those reported previously that *OsARF19* and *OsIAA1* positively regulate leaf inclination, and *OsTIR1* negatively regulates leaf inclination (Song et al., 2009; Bian et al., 2012; Zhang et al., 2015), implying that *OsARF4* might play a negative role in regulating leaf inclination.

### Increased cell proliferation on the adaxial surface leads to increased leaf inclination in *osarf4*

**Figure 3:**
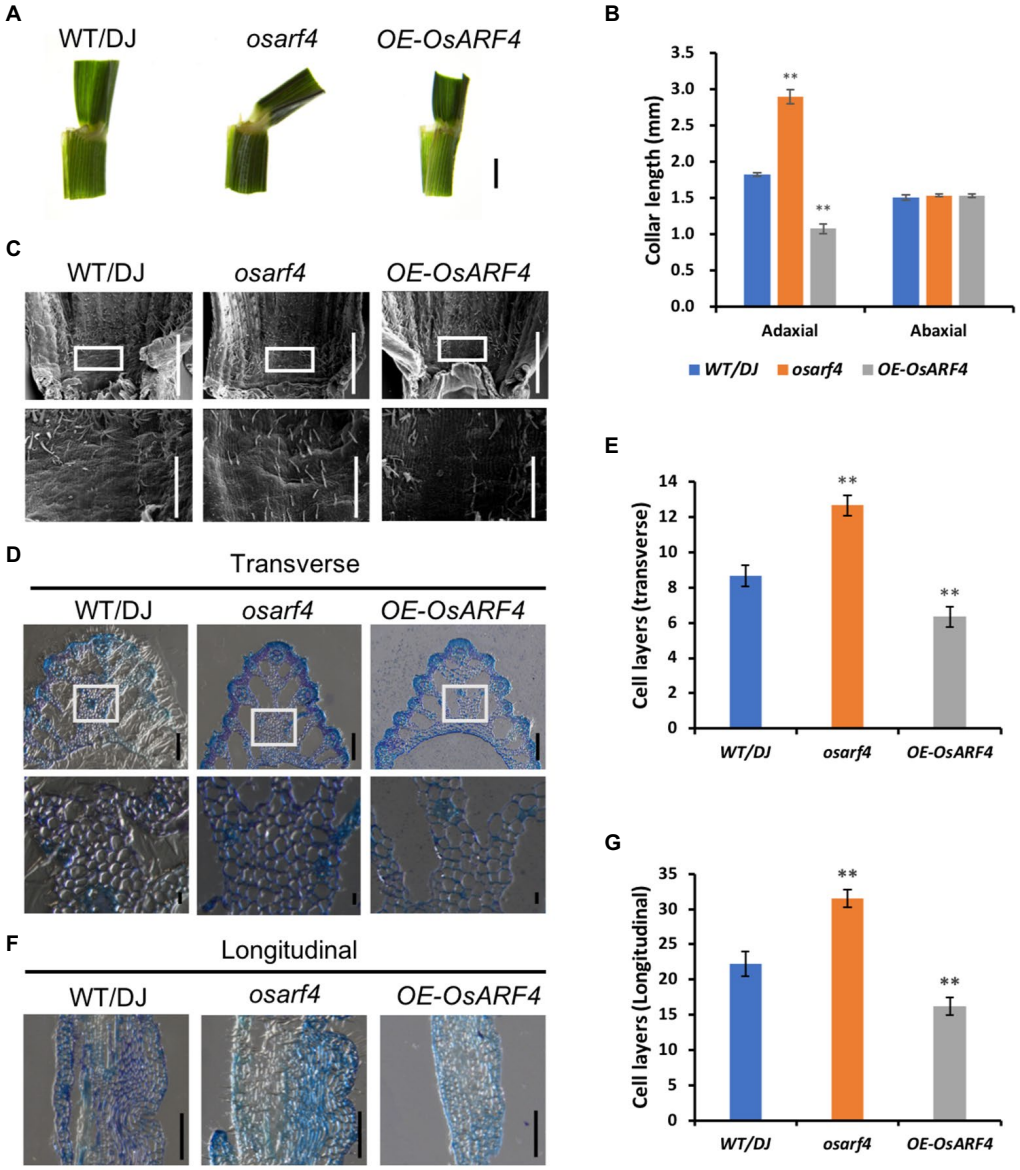
Microstructure analysis of LJs in WT/DJ, *osarf4* mutants, and *OsARF4-*overexpressing lines. **(A)** Comparison of LJs of flag leaves in 3-month-old seedlings of WT/DJ, *osarf4* mutants, and *OsARF4-*overexpressing lines. **(B)** The collar length of the flag leaves at adaxial and abaxial surfaces in the above-mentioned lines. **(C)** SEM images of the adaxial surface of the LJ of the flag leaves. **(D)** Transverse (cross) section of LJs of flag leaves in the above-mentioned lines. Upper panel, bar = 200 μm; lower panel shows the magnified parts of the upper panel, bar = 20 μm. **(E)** Cell layers in transverse sections. Ten biological replicates were considered; **p < 0.01 represents significant differences using Student’s *t*-test. **(F)** Longitudinal section of LJs of flag leaves in the above-mentioned lines. Bar = 200 μm. **(G)** Cell layers in longitudinal section of LJs. Ten biological replicates were considered for each test. **p < 0.01 indicates a significant difference compared with WT/DJ using Student’s *t*-test.

Previous studies have shown that the LJ significantly contributes to the formation of leaf inclination, and the increased leaf inclination may cause alterations in LJ development (Yamamuro et al., 2000). Therefore, the collar length of the LJ at adaxial and abaxial epidermis in WT/DJ, *osarf4* mutants, and *OsARF4*-overexpressing lines was measured. As shown in Figures 3A,B, the collar length of the LJ at adaxial surface of *osarf4* mutants increased by 60%, whereas it decreased by 45% in *OsARF4*-overexpressing lines compared with that in WT/DJ. In contrast, there were no significant differences in the length at the abaxial surface between WT/DJ and *osarf4* mutants or *OsARF4*-overexpressing lines. These results indicate that the main difference in leaf inclination in those lines was due to the imbalanced elongation between the adaxial and abaxial surfaces of LJ.

To identify the cellular mechanism underlying the regulation of leaf inclination by *OsARF4*, SEM analysis was conducted to observe microstructure morphology. In *osarf4* mutant, there was much larger and formed a bulge in the adaxial surface of the LJ, whereas it was smoother in *OsARF4*-overexpressing lines compared to the smooth adaxial surface in WT/DJ (Figure 3C). These results further confirmed that the development of the LJ in *osarf4* was altered and resulted in increased leaf inclination.

Previous studies have shown that aberrant cellular development, such as lack of the longitudinal cell elongation or increase in cell expansion at the adaxial sides in the collar, can lead to changes in leaf inclination (Duan et al., 2006; Zhao et al., 2010; Zhang et al., 2015; Guo et al., 2021; Tian et al., 2021). To further reveal the reason for the changed leaf inclination in the mutants and overexpression lines of *OsARF4*, we prepared paraffin sections with both transverse and longitudinal sections of the LJs of WT/DJ, *osarf4* mutants, and *OsARF4*-overexpressing lines. The results indicate that the number of sclerenchyma cell layers on the adaxial surface increased in both transverse and longitudinal sections in *osarf4* mutants and decreased in the *OsARF4*-overexpressing lines, compared with that in WT/DJ (Figures 3D–G), which illustrates that cell division, but not cell elongation, in the LJ resulted in increased leaf inclination in *osarf4*.

### *OsARF4* regulates leaf inclination by controlling auxin distribution and content in lamina joints

Auxin is an important plant hormone that plays a negative role in controlling leaf inclination (Du et al., 2012; Zhou et al., 2017). Studies have shown that *OsARF* regulates leaf angles induced by auxin (Zhang et al., 2015). To explore the role of auxin in *OsARF4*-mediated leaf inclination pathway, the relative expression level of *OsARF4* was detected under auxin treatment. It was found that auxin treatment could induce *OsARF4* expression (Supplementary Figure 3), indicating that the function of *OsARF4* might be positively regulated by auxin.

**Figure 4:**
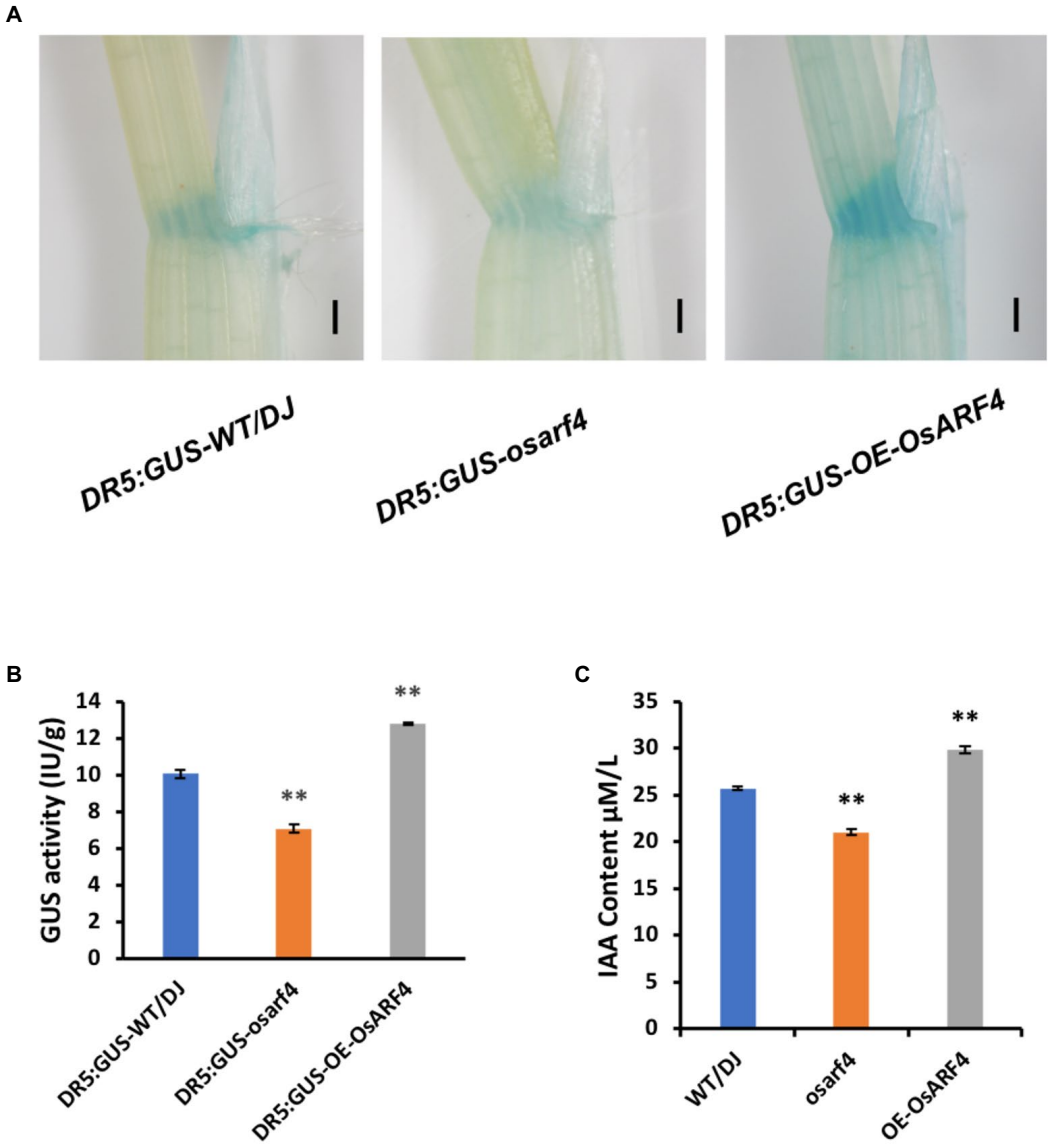
Analysis of auxin distribution and content in LJs in WT/DJ, *osarf4* mutants, and *OsARF4-*overexpressing lines. **(A)** GUS staining of *DR5:GUS-WT/DJ*, *DR5:GUS-osarf4*, and *DR5:GUS-OE-OsARF4* transgenic lines in the LJs. Bars = 500 μm. **(B)** Quantification of GUS activity in *DR5:GUS-WT/DJ*, *DR5:GUS-osarf4*, and *DR5:GUS-OE-OsARF4* transgenic lines. Ten biological replicates were evaluated. **p < 0.01 indicates a significant difference in means ± SD compared with *DR5:GUS-WT/DJ* based on Student’s *t-test*. **(C)** Determination of auxin contents in LJs of WT/DJ, *osarf4* mutants, and *OsARF4-*overexpressing lines. Five separate biological replicates were used in each test. **p < 0.01 indicates significant differences from WT/DJ based on Student’s *t-test*.

As the auxin response reporter, *DR5:GUS* has been widely used to study the distribution of endogenous auxin (Ulmasov et al., 1997; Qiao et al., 2021). To determine whether the changes in leaf inclination of *OsARF4* are influenced by the changes in auxin distribution, *DR5:GUS* was transformed into the *osarf4* mutants and *OsARF4*-overexpressing lines. GUS staining in LJs indicated that *DR5:GUS* expression in *osarf4* mutants was markedly lower than that in WT/DJ but more intense in *OsARF4*-overexpressing lines (Figure 4A). Moreover, *GUS* activity was lower in *osarf4* but higher in *OsARF4*-overexpressing lines, compared with that in WT/DJ (Figure 4B). Furthermore, the free auxin content in LJs of WT/DJ, *osarf4* mutants, and *OsARF4*-overexpressing lines was measured. The results showed that the auxin content was lower and higher in *osarf4* mutants and *OsARF4*-overexpressing lines, respectively, compared with that in WT/DJ (Figure 4C), implying that *OsARF4* regulates leaf inclination by controlling local auxin levels in LJs.

### RNA-seq analysis of WT/DJ, mutant and overexpression line of *OsARF4*

**Figure 5:**
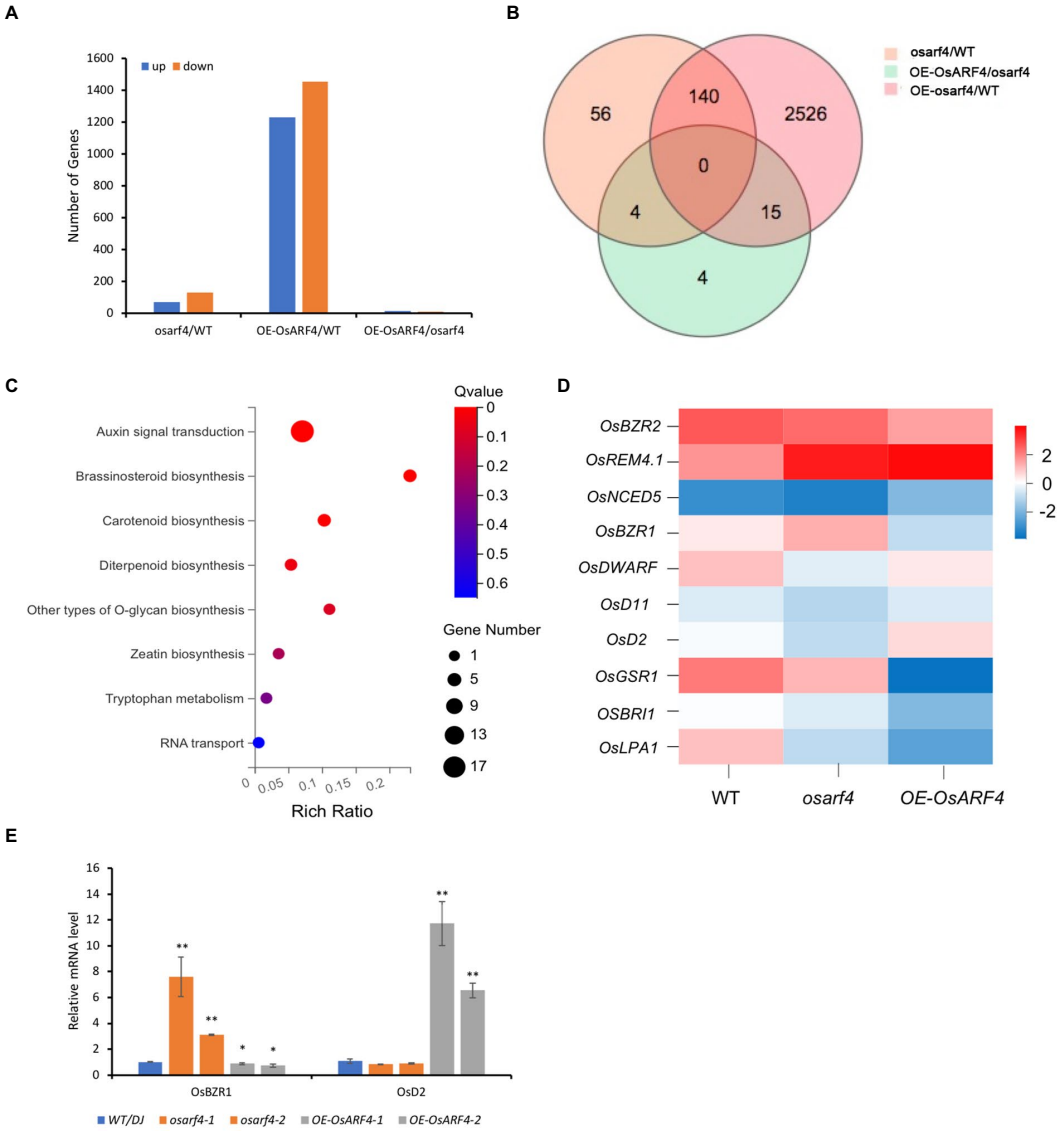
RNA-seq analysis of *OsARF4*-modulated genes in rice LJs. **(A)** Numbers of DEGs in the LJs of WT/DJ versus *osarf4*, WT/DJ versus *OE-OsARF4,* and *osarf4* versus *OE-OsARF4*. Blue columns represent up-regulated genes, and orange columns represent downregulated genes. **(B)** Venn diagram showing an overlap of DEGs in LJs in WT/DJ versus *osarf4*, WT/DJ versus *OE-OsARF4,* and *osarf4* versus *OE-OsARF4* in LJs. **(C)** GO enrichment functional clustering of *OsARF4*-modulated genes that were differentially expressed in the transcriptome. X-axis represents enrichment ratio. The size of bubbles represents the number of DEGs annotated to a certain GO term. The color represents enrichment significance value. **(D)** Heat map of the RNA-seq analysis revealed the expression patterns of selected genes during BR biosynthesis and signaling in WT/DJ, *osarf4* mutant, and *OsARF4*-overexpressing lines. **(E)** Relative expression of *OsBZR1* and *OsD2* in two *osarf4* mutants and two *OE-OsARF4* lines. Three individual biological replicates were taken for each experiment. *p < 0.05 and **p < 0.01 indicate significant differences compared with WT/DJ using Student’s *t*-test.

To analyze genes of *OsARF4*-mediated pathway that participate in the regulation of leaf inclination, transcriptome of the flag LJs from WT/DJ, *osarf4* mutant, and *OsARF4*-overexpressing line was sequenced. A total of 32,938 genes were obtained in the RNA-seq analysis. Among those differentially expressed genes (DEGs), 70 genes were upregulated and 130 genes were downregulated between WT and *osarf4* mutant; 1,229 genes were upregulated and 1,452 genes were downregulated between the WT and *OsARF4*-overexpressing lines; and 12 genes were upregulated and 11 genes were downregulated between the *osarf4* mutant and *OsARF4*-overexpressing line (Figures 5A,B). The results of gene ontology (GO) enrichment analysis demonstrated that genes related to auxin signal transduction and BR biosynthesis were significantly overrepresented (Figure 5C), which implies that *OsARF4* might be involved in auxin signal transduction and BR biosynthesis. In previous studies, genes related to regulation of leaf inclination, including *OsBZR2* (Min et al., 2019), *OsREM4.1* (Li et al., 2021), *OsNCED5* (Zhu et al., 2009), *OsBZR1* (Bai et al., 2007), *OsDWARF* (Hong et al., 2002), *OsD11* (Tanabe et al., 2005), *OsD2* (Hong et al., 2003) *OsGSR1* (Wang et al., 2009), *OsBRI1* (*Brassinazole-resistant 1*; Yamamuro et al., 2000), and *OsLPA1* (Wu et al., 2013), have been reported, and these genes showed variable expression in LJs of *osarf4* mutant and *OsARF4*-overexpressing line compared with that in WT/DJ (Figure 5D and Supplementary Figure 4). These results indicate the importance of *OsARF4* in BR signaling. Furthermore, as shown in Figure 5D, the RNA-seq results indicated that the expression of *OsBZR1* is upregulated in *osarf4* mutant and downregulated in *OE-OsARF4* line. On the contrary, the expression of *OsD2* is downregulated in *osarf4* mutant and upregulated in *OE-OsARF4* line. To confirm the results, qRT-PCR was performed in the flag leaf of WT/DJ, *osarf4* mutants, and *OE-OsARF4* lines. The results were consistent with the RNA-seq analysis (Figures 5D,E). *OsBZR1* is a famous gene associated with brassinosteroid signal transduction in rice (Bai et al., 2007). *OsD2* encodes a cytochrome P450 BR synthesis enzyme in the late BR biosynthesis pathway (Hong et al., 2003). Overall, whether *OsBZR1* and *OsD2* are the potential target genes of *OsARF4* needs to be further explored.

### *OsARF4* is sensitive to exogenous BR

**Figure 6:**
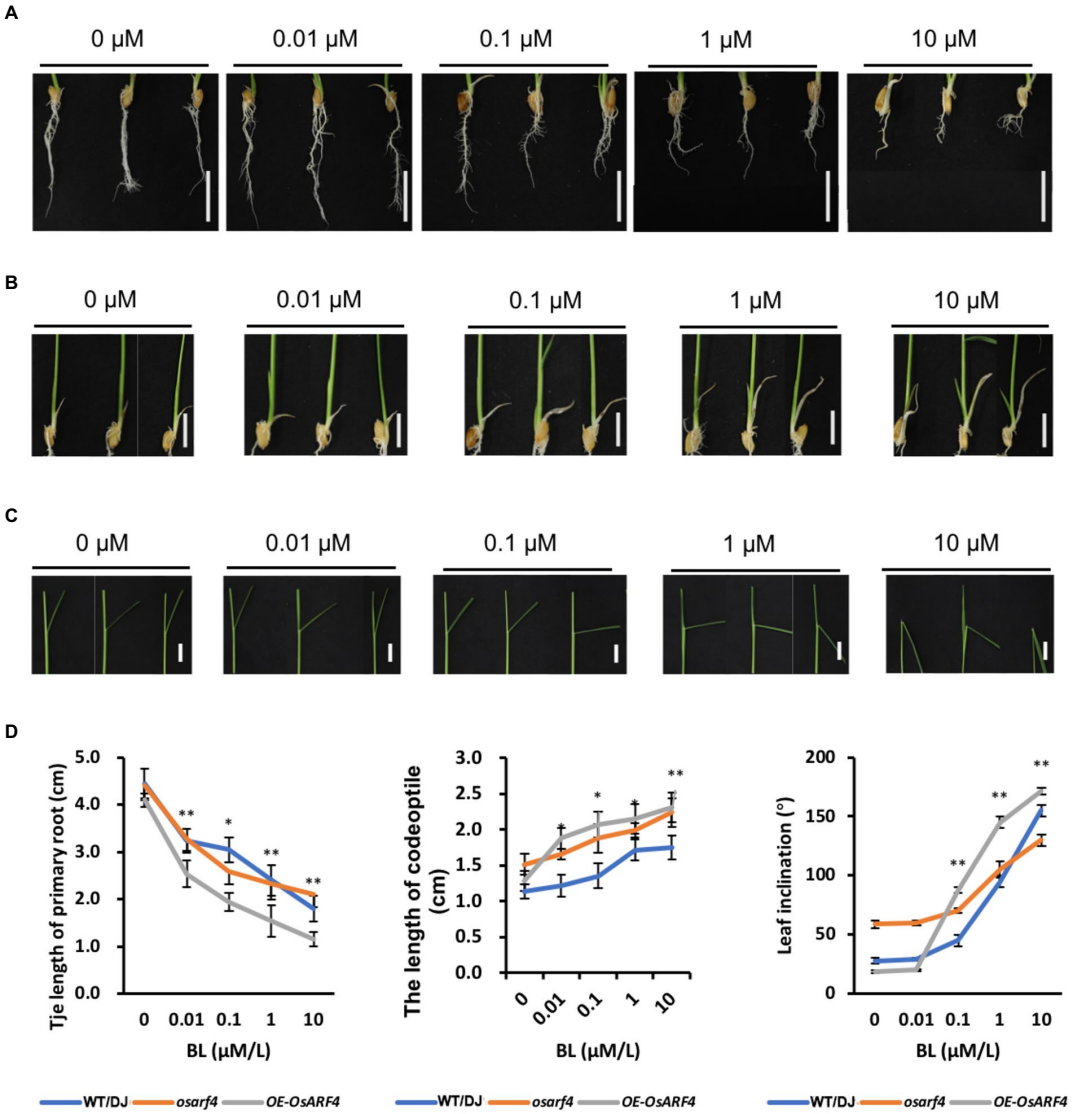
*OsARF4* mediates the morphological response of rice to 24-epibrassinolide (24-eBL). **(A–C)** primary root (PR; **A**), coleoptile elongation **(B)**, and Leaf inclination **(C)** response to 24-eBL treatments. Panels from left to right show roots of seedlings of WT/DJ, *osarf4* mutants, and OsARF4-overexpressing lines grown for 7 days **(A,B)** or 3 days **(C)** under 0, 0.01, 0.1, 1, and 10 μm 24-eBL treatment. Bars = 2 cm in **A** and 1 cm in **B/C**. **(D)** Statistical analyses of PR length, coleoptile, and leaf inclination in WT/DJ, *osarf4* mutants, and *OsARF4*-overexpressing lines under above indicated 24-eBL treatments (*n* = 10). *p < 0.05 and **p < 0.01 based on Student’s *t-test*.

In rice, BR has been proved to play a pivotal role in the determination of leaf inclination (Sakamoto et al., 2013; Zhang et al., 2015). To further confirm whether *OsARF4* is involved in BR signaling in regulation of leaf inclination, the three classical BR sensitivity experiments were performed. In addition to LJs, *OsARF4* was also expressed in primary root, lateral root, and coleoptile (Figure 1A; Supplementary Figure S5). Therefore, BR sensitivity experiments, PR elongation, coleoptile elongation, and the degree of leaf inclination tests were performed under treatments with different concentrations of 24-eBL (Figure 6). The PR growth was inhibited by BR treatments and this inhibition increased with the increased in BR concentration. Compared to the WT/DJ, *osarf4* mutants were insensitive to exogenous BR treatments, whereas *OsARF4*-overexpressing lines were relatively more sensitive (Figures 6A,D). After BR treatments, the elongation of coleoptile increased in *OsARF4*-overexpressing lines and became more pronounced with the increase in BR concentration, whereas *osarf4* mutants showed slight increase compared with that in WT/DJ (Figures 6B,D). The leaf inclination in *OsARF4*-overexpressing lines and *osarf4* mutants increased by 8–80% and 3–20%, respectively, under treatments with different concentrations of 24-eBL (Figures 6C,D). Taken together, the *OsARF4*-overexpressing lines were more sensitive to exogenous BR treatments, whereas the *osarf4* mutants were insensitive, compared with WT/DJ.

**Figure 7:**
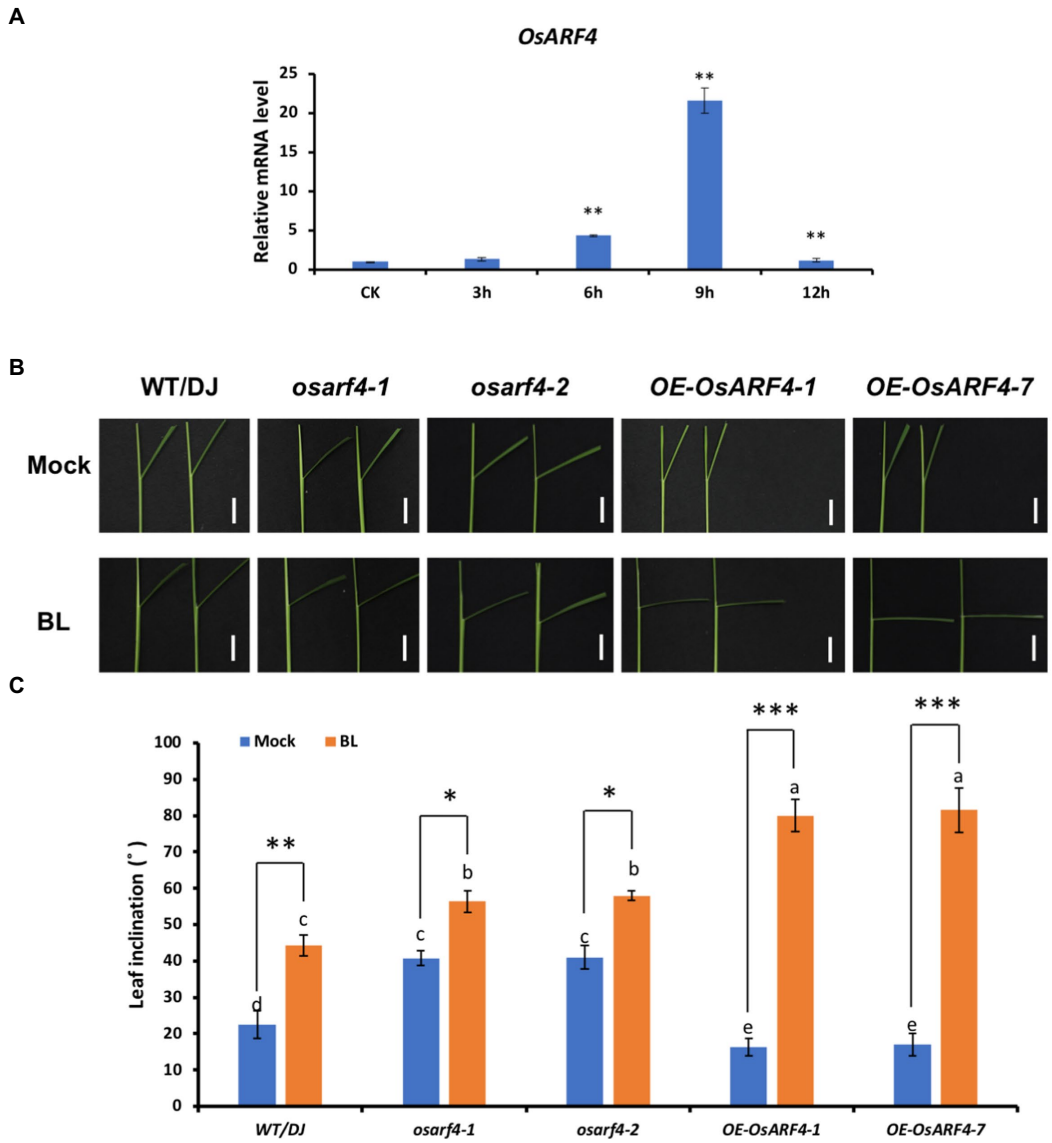
*OsARF4* is sensitive to low concentration of BR in increasing leaf inclination of rice seedling. **(A)** The expression of *OsARF4* under 0.1 μm 24-eBL treatment at different time intervals. **(B)** Leaf inclination response of 7 days seedlings of WT/DJ, *osarf4-1*, *osarf4-2*, *OE-OsARF4-1*, and *OE-OsARF4-7* to 0.1 μm 24-eBL treatment for 3 days. **(C)** Measurement of leaf inclination in response to 0.1 μm 24-eBL treatment for 3 days as shown in **B**. Fifteen rice lines were measured for each treatment. Error bars indicate SD (*n* = 3). Lowercase letters represent significance based on Duncan’s multiple range test (α = 0.05). *p < 0.05, **p < 0.01 and ***p < 0.001 indicate significant differences of means ± SD compared with control based on *t*-test.

Moreover, *OsARF4* expression was induced by BR treatments (Figure 7A). When *osarf4* mutants and *OsARF4*-overexpressing lines were treated with 0.1 μM 24-eBL (the optimum concentration selected from the above gradient treatment) for 3 days simultaneously, the leaf inclination of WT/DJ, *osarf4* mutants, and *OsARF4*-overexpressing lines increased by 96, 38, and 390%, respectively (Figures 7B–C). Hence, *OsARF4*-overexpressing lines were more sensitive, whereas *osarf4* mutants were insensitive to BR treatments, compared with WT/DJ. These results suggest that *OsARF4* is related to regulation of BR signaling-mediated leaf inclination in rice.

## Discussion

### *OsARF4* functions in regulating leaf inclination

As plant-specific B3-type transcription factors, ARFs play a role in activating or repressing the auxin response genes by specifically binding to auxin response element (AuxRE; Chandler, 2016). There are 25 *ARFs* in rice, many of which have been reported to execute multiple functions (Wang et al., 2007). For instance, *OsARF12* and *OsARF16* are associated with root development and phosphate homeostasis (Qi et al., 2012; Shen et al., 2013, 2014; Wang et al., 2014). *OsARF25* is involved in controlling grain size, and its T-DNA insertion mutant has shorter grains (Zhang et al., 2018). *OsmiR160* negatively regulates the expression of *OsARF18* and improves rice growth and development *via* auxin signaling pathway (Huang et al., 2016a). *OsARF6* participates in the *miR167a-OsARF6-OsAUX3* module to regulate grain length and grain weight in rice (Qiao et al., 2021). *OsARF6* and *OsARF17* bind to the promoter of *ILA1* to determine flag leaf angle (Huang et al., 2021). *OsARF1* interacts with OsIAA6 to regulate leaf inclination in rice through auxin and BR pathway (Xing et al., 2022). In a previous study, the research of our laboratory have found that *OsARF19* is involved in positive regulation of lamina inclination regulation by binding to the promoters of *OsGH3-5* and *OsBRI1*, through auxin and BR pathway (Zhang et al., 2015). In this study, we generated two *osarf4* mutants using CRISPR/Cas9 gene editing technology, and two *OsARF4*-overexpressing lines using genetic transformation. Phenotype analysis showed that the flag leaf inclination increased by approximately 150% in *osarf4* mutants and decreased by 44% in *OsARF4*-overexpressing lines after heading in rice (Figure 2; Supplementary Figure 2). Leaf inclination is mainly determined by the LJ, which is a unique tissue connecting the leaf blade and sheath in rice (Wang et al., 2020). GUS staining and qRT-PCR results indicated that *OsARF4* was expressed in LJ at different developmental stages (Figures 1A,B), thus confirming that *OsARF4* is involved in leaf inclination regulation. Interestingly, both *OsARF4* and *OsARF19* belong to the *OsARF* family, but they play converse roles in the regulation of leaf inclination in rice, because they were from different clades, namely, *OsARF4* belongs to Class I with transcript repression domain while *OsARF19* belongs to Class II with transcript activation domain (Wang et al., 2007; Shen et al., 2010), lead to they have different functions in leaf inclination regulation.

From a cytological perspective, the change in leaf inclination is attributed to the imbalance in the cell development of the adaxial and abaxial sides in LJs (Duan et al., 2006). In previous studies, most of the identified rice mutants with altered leaf inclination showed abnormal division and expansion of adaxial cells in the leaf collar (Nakamura et al., 2009; Zhang S. W. et al., 2009; Zhao et al., 2010; Ning et al., 2011). The leaf inclination is caused by the bending of the blade. From the perspective of a cytological structure, the most obvious change is the cell division and expansion at the adaxial side of the LJ (Wang et al., 2020). To illustrate the cytological structures of mutants and overexpression lines of *OsARF4*, the collar lengths in the adaxial and abaxial sides were measured. The results demonstrated that the asymmetric collar length in LJs alters the leaf inclination in the mutants and overexpression lines of *OsARF4*. Furthermore, according to the cytological structural analysis of LJs, it was found that *OsARF4* regulates leaf inclination through modulating adaxial cell division rather than cell expansion of the collar in LJs (Figure 3). The increase of leaf inclination in *osarf4* is achieved by promoting the differentiation of the adaxial side parenchyma cells, which is mainly to promote the synthesis of genes related to cell wall such as the cyclin gene family members. The cell cycle-related genes *OsCYCU4;1*, *OsCYCD2;1, OsCYCD6;1*, and *OsCYCD1;2* also changed differently in the RNA-seq analyses of WT/DJ, *osarf4* mutant, and *OsARF4*-overexpressing line in LJs (Supplementary Figure 6). In addition, whether auxin has polar distribution in the adaxial side and abaxial side of LJs needs to be further explored.

### The change in auxin level in lamina joint might alter leaf inclination

Auxin responses play a crucial role in plant growth and development by forming local concentration gradients. Auxin induces rapid transcriptional responses, incorporating a series of auxin early response genes (*SAUR, Aux/IAA,* and *GH3*) and ARFs. It is known that auxin synthesis and signal transduction are related to the regulation of leaf inclination (Xu et al., 2021). Moreover, during early LJ development, high local auxin concentrations were formed due to auxin synthesis and polar transport, which will stimulate cell division. During LJ development, a decrease in IAA content will promote the elongation of parenchyma cells on the adaxial side of the LJ, which is consistent with the increase in *GH3* expression, thus increasing leaf inclination (Wang et al., 2007; Chen et al., 2018). The auxin signaling pathway is normally mediated by *ARFs* that regulate transcription in response to auxin in plants (Guilfoyle and Hagen, 2007). In this study, we have shown that *OsARF4* regulated leaf inclination *via* altering the auxin distribution and content in LJ. As shown in Figure 4, the auxin distribution and GUS activity decreased in *osarf4* mutants, they significantly increased in *OsARF4*-overexpressing lines compared with those in WT/DJ. The free auxin levels in LJs were consistent with this result, implying that the alteration of auxin level in LJ regulated by *OsARF4* results in changes in leaf inclination. The RNA-seq analyses also revealed that some auxin-catabolism and auxin signaling-related genes such as *OsGH3-1*, *OsGH3-2*, *OsGH3-5*, *OsAFB2*, *OsARF1,* and *OsIAA1* showed different expression levels in the LJs of WT/DJ, *osarf4* mutant, and *OsARF4*-overexpressing line (Supplementary Figure 6).

### *OsARF4* mediates auxin-BR crosstalk during regulation of leaf inclination

Auxin and BR are two vital phytohormones that regulate leaf inclination in rice (Tong and Chu, 2018). Studies have shown that lacking of BR leads to an erect leaf phenotype because of the inhibition of elongation of parenchyma cells and division of sclerenchyma cells on the abaxial side of the LJ (Zhang L. Y. et al., 2009; Sun et al., 2015). Most BR-deficient and BR-insensitive mutants show altered BR sensitivity by altering auxin synthesis or signal transduction, and many auxin-related mutants also show altered BR responses by changing leaf inclination, thus suggesting crosstalk between BR and auxin during LJ development (Song et al., 2009; Zhao et al., 2013; Zhang et al., 2015). The signaling pathways of auxin and BR share multiple genes, which play important roles in plant growth and development (Vert et al., 2008). In rice, *OsARF19* regulates leaf inclination by binding to the promoter region of *OsBRI1* to connect auxin and BR signaling pathways (Zhang et al., 2015). The *ds1* mutant shows decreased leaf angle owing to lower BR sensitivity, and *DS1* participates in plant architecture regulation through regulating the expression of *OsBRI1 via* interacting with *OsARF11* in rice (Liu et al., 2018). Therefore, BR and auxin genes act synergistically to co-regulate the development of adaxial cells in LJ (Liu et al., 2016). In this study, both auxin and BR induced the expression of *OsARF4* (Figure 7A; Supplementary Figure 3). DR5:GUS staining and measurement of free IAA content showed that *OsARF4* is involved in auxin signaling (Figure 4). RNA-seq analysis indicated that *OsARF4* is related to BR biosynthesis and signaling (Figure 5). Furthermore, *OsARF4-*overexpressing lines were sensitive to BR treatments and had increased leaf inclination (Figures 6, 7B–D). These results indicate that *OsARF4* acts as a bridge between auxin and BR signaling during the regulation of leaf inclination.

In addition, a previous study has reported that OsARF4 interacts with the rice GSK3-like kinase, OsGSK5/OsSK41, to negatively regulate grain size and weight in rice. And, *osarf4* mutant was no effect on BR sensitivity in terms of leaf lamina inclination at the seedling stage (Hu et al., 2018). In this study, we observed that *osarf4-1* and *osarf4-2* had enlarged leaf inclination at the flag leaf of mature period (Figures 2C–D). As for BR sensitivity, the BR sensitivity experiments were treated with different concentrations 0, 0.01, 0.1, 1, and 10 μm of 24-eBL. The above tests presented that *osarf4* mutants are insensitive to exogenous BR treatments, while *OsARF4*-overexpressing lines are more sensitive not only in the degree of leaf inclination but also in PR elongation and coleoptile growth (Figure 6). Besides, the different editing sites and background plants from previous research might result in a little difference of regulatory mechanism (Hu et al., 2018).

Taken together, the semi-dwarf phenotype, characterized by erect leaves and shorter panicles, is an ideal phenotype for improving grain yield in high-density planting in rice (Morinaka et al., 2006; Sakamoto et al., 2006; Wu et al., 2008). Therefore, the regulation mechanism of leaf inclination can provide a theoretical basis for plant architecture breeding that would help develop highly productive rice varieties. In our study, evidence from genetic analysis, cellular biological observation, molecular biological analysis, and physiological experiments support the finding that *OsARF4* regulates leaf inclination *via* auxin and BR signaling pathways in rice. Improving plant architecture is an important goal for breeding of high-yield rice varieties. Erect leaves can increase planting density, improve photosynthetic capacity, increase accumulation of photosynthetic products, and ultimately increase the grain yield of rice (Huang et al., 2016b). Our research uncovers a normal regulatory factor of leaf inclination and shows that a molecular bridge exists between auxin and BR signaling. These findings may help optimize plant architecture and increase yield of rice.

## Data availability statement

The data presented in the study are deposited in the NCBI repository, accession number PRJNA862303.

## Author contributions

YQ and QQ designed the experiments. JQ, YZ, SH, and SC performed experiments. YQ, QQ, ZG, and JQ analyzed the data. YQ, QQ, and JQ wrote the manuscript. All authors contributed to the article and approved the submitted version.

## Funding

This project was funded by grants from the National Natural Science Foundation of China (32060451), the Zhejiang Provincial Natural Science Foundation of China (grant no. LZ19C020001), Natural Science Foundation of Inner Mongolia (2022ZD11), and Applied Technology Research and Development Foundation of Inner Mongolia (2021PT0001) to YHQ, the Special Fund of Hainan Yazhou Bay Seed Laboratory to JYQ (B21Y10202), and China Postdoctoral Science Foundation (2022T150714).

## Conflict of interest

The authors declare that the research was conducted in the absence of any commercial or financial relationships that could be construed as a potential conflict of interest.

## Publisher’s note

All claims expressed in this article are solely those of the authors and do not necessarily represent those of their affiliated organizations, or those of the publisher, the editors and the reviewers. Any product that may be evaluated in this article, or claim that may be made by its manufacturer, is not guaranteed or endorsed by the publisher.

## Footnotes

^1^ http://www.ruixinbio.com/

^2^ https://www.Genomics.cn/

## Supplementary material

SUPPLEMENTARY FIGURE 1 Nuclear localization of OsARF4 in root cells. *35S:OsARF4-GFP* was transformed into WT/DJ. Green fluorescence represents the nucleus of the root tip cell; bar scale = 20 μm.

SUPPLEMENTARY FIGURE 2 Relative expression of *OsARF4* in *OsARF4*-overexpressing lines. qRT-PCR was performed using the LJs of each line for three individual biological replicates. ^*^*P* < 0.05 and ^**^*P* < 0.01, *t*-test.

SUPPLEMENTARY FIGURE 3 *OsARF4* expression under auxin treatments. Seven-day-old of WT/DJ seedlings were grown in a solution containing 10 μm NAA and 1 μm 2,4-D for 0, 3, 6, 9, and 12 h. (*n* = 3) ***P* < 0.01 indicates significant differences compared with 0 h using Student’s *t*-test.

SUPPLEMENTARY FIGURE 4 Expression profiles of hormone-related genes in RNA-seq in LJs of WT/DJ, *osarf4*-1 and *OE-OsARF4-1* line.

SUPPLEMENTARY FIGURE 5 *OsARF4* is expressed in primary root, lateral root and coleoptile of *pro:OsARF4-GUS* transgenic seedlings. **(A)** GUS staining of 3-d-old primary root, lateral root and coleoptile of *pro:OsARF4-GUS* transgenic seedlings. Bar scale = 500 μm. **(B)** qRT-PCR analysis of *OsARF4* expression in 3-d-old primary root, lateral root and coleoptile. Error bars represent SD (*n* = 3). Lowercase letters represent significance according to Duncan’s multiple range test (α = 0.05).

SUPPLEMENTARY FIGURE 6 Heat map of the RNA-seq analysis of genes involved in cell division and auxin signaling in LJs.

SUPPLEMENTARY TABLE 1 Primers used for vector construction.

SUPPLEMENTARY TABLE 2 Primers used for qRT-PCR.
