# Supplementary material for: OsARF4 regulates leaf inclination via auxin and brassinosteroid pathways in rice
Source: Front Plant Sci. 2022 Sep 30;13:979033. doi: 10.3389/fpls.2022.979033 (PMC9561258; doi:10.3389/fpls.2022.979033)
Supplement: SUPPLEMENTARY FIGURE 1 — Nuclear localization of OsARF4 in root cells. 35S:OsARF4-GFP was transformed into WT/DJ. Green fluorescence represents the nucleus of the root tip cell; bar scale = 20 μm. [file Data_Sheet_1.pdf]

Supplementary Figures

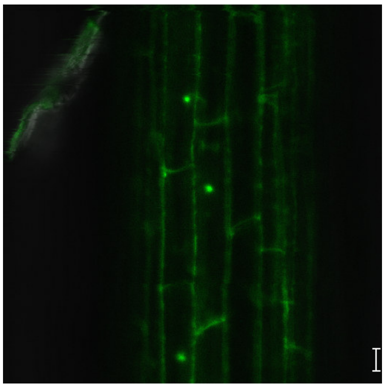

Supplementary Figure 1. Nuclear localization of OsARF4 in root cells.

35S:OsARF4-GFP was transformed into WT/DJ. Green fluorescence represents the nucleus of the root tip cell; bar scale = 20 μm.

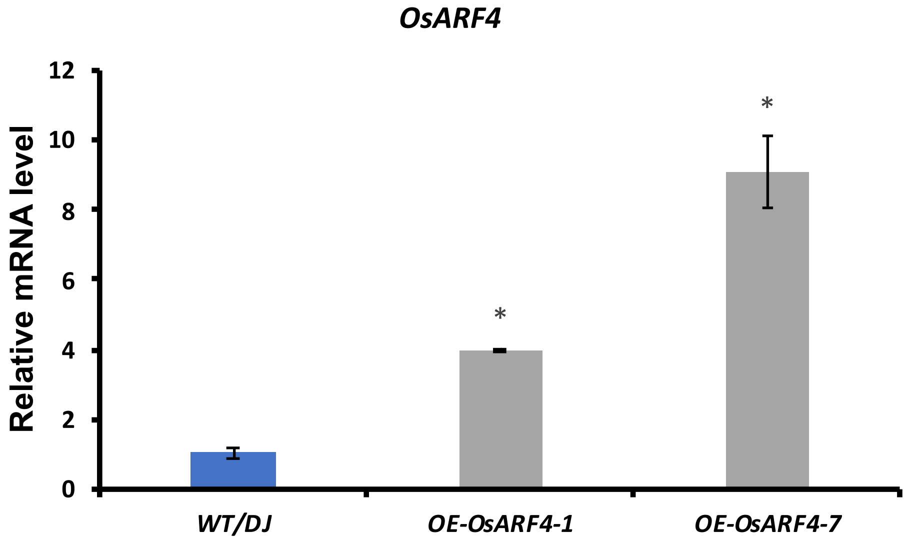

Supplementary Figure 2. Relative expression of *OsARF4* in *OsARF4*-overexpressing lines.

qRT-PCR was performed using the LJs of each line for three individual biological replicates. *$P < 0.05$, *t*-test.

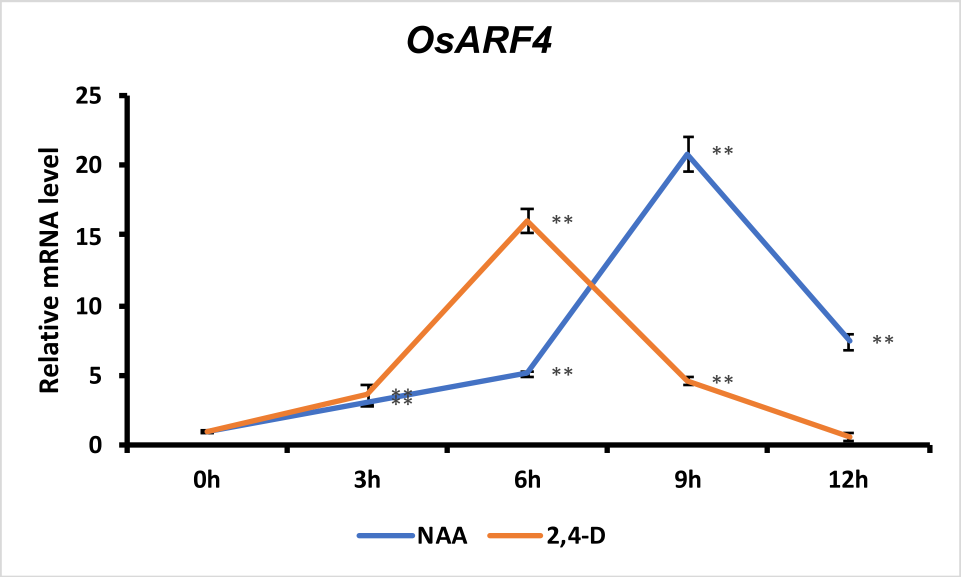

Supplementary Figure 3. *OsARF4* expression under auxin treatments.
Seven-day-old of WT/DJ seedlings were grown in a solution containing 10 μM NAA and 1 μM 2,4-D for 0 h, 3 h, 6 h, 9 h, and 12 h. (n = 3) **P < 0.01 indicates significant differences compared with 0 h using Student's t-test.

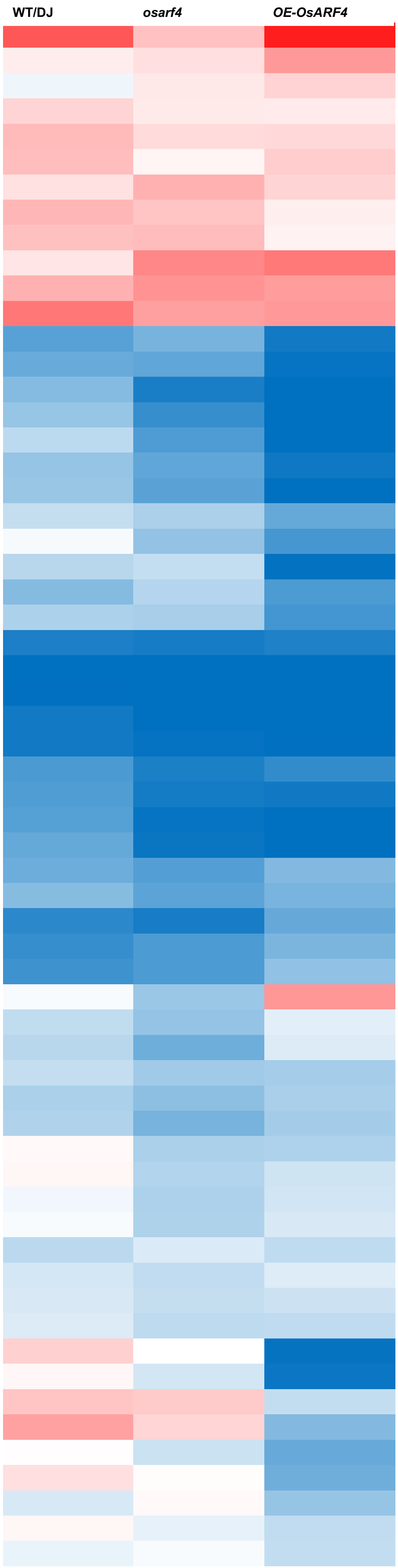

Genw name	Gene ID
ABA1	4335984
OSE2	4325078
IAA6	4326541
SPY	4346315
AGO4B	4335025
GH3.5	4339756
MADS55	4340495
BZR2	4327076
OsJ_14626	4335696
OJ1019_E02.9	4343649
MADS22	4330805
AGO4A	4324438
LG1	4337258
IAA12	4333512
AS2	4324906
IAA31	4352722
BHLH172	4340551
GH3.11	4344247
IAA14	4334431
BRI1	4324691
Lpa1	4331161
BHLH154	107278038
PIN1	4330700
ARF8	4330045
OJ1123_G04.23	4330337
LAX1	4327431
OsVP1	4324314
GH3.1	4327043
AGO7	4333232
DLT	4339978
D18	4323864
PGL1	4331776
OREB1	4325061
ABA8ox2	4345810
ESG1	4326709
NCED5	9270250
ZHD1	4347315
CKX2	4327333
GA20ox2	4325003
D2	4327329
TAR2	4325198
RDR2	4336092
DWARF11	4336116
GH3.2	4326893
IAA1	4323871
MADS4	4331872
ARF1	4327785
CYP85A1	4333399
GA2ox3	4325145
BHLH175	4337280
C3H46	4341994
DCL3A	4324864
Gsr1	4340712
IAA30	4352721
OsJ_04119	4324749
ARF12	4337363
IAA18	4339365
ARF17	4341841
BZR1	4343719
ARF25	4352783
ARF3	4327014

Supplementary Figure 4. Expression profiles of hormone-related genes in RNA-seq in LJs of WT/DJ, *osarf4*-1 and *OE-OsARF4-1* line.

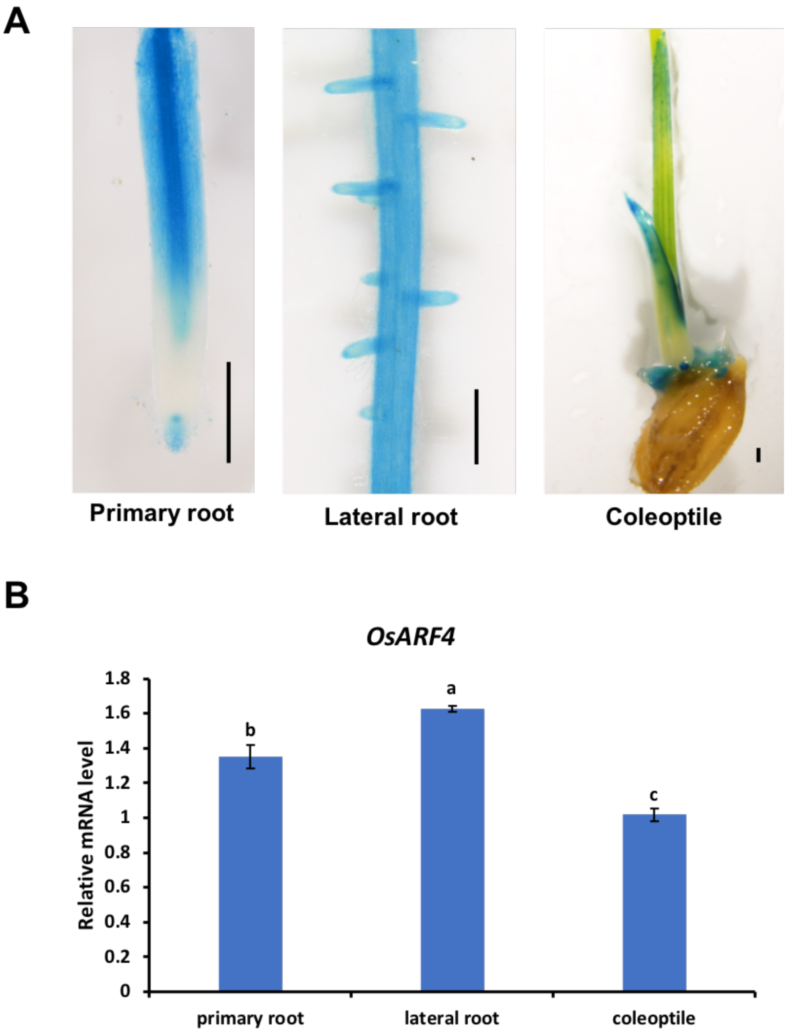

Supplementary Figure 5. *OsARF4* is expressed in primary root, lateral root and coleoptile of *pro:OsARF4-GUS* transgenic seedlings. (A) GUS staining of 3-d-old primary root, lateral root and coleoptile of *pro:OsARF4-GUS* transgenic seedlings. Bar scale = 500 μm. (B) qRT-PCR analysis of *OsARF4* expression in 3-d-old primary root, lateral root and coleoptile. Error bars represent SD (n = 3). Lowercase letters represent significance according to Duncan's multiple range test (α = 0.05).

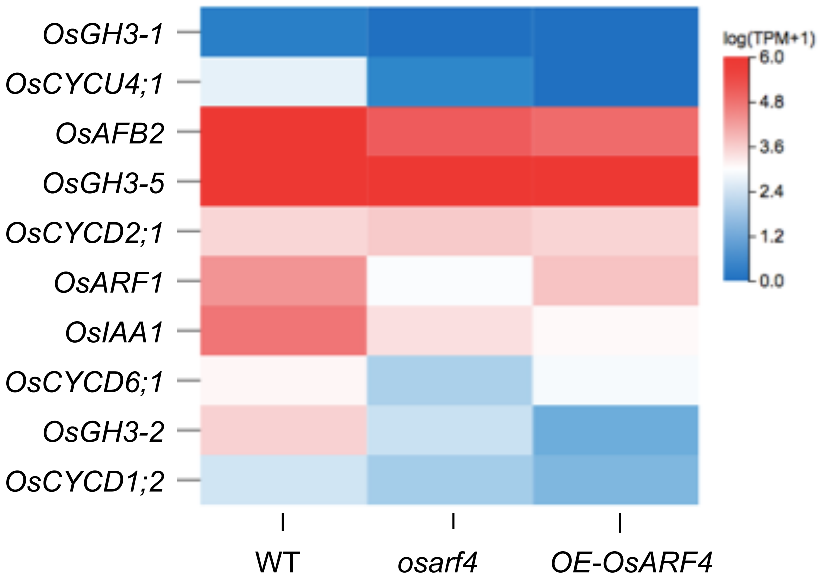

Supplementary Figure 6. Heat map of the RNA-seq analysis of genes involved in cell division and auxin signaling in LJs.

Supplementary Tables

Supplementary Table 1. Primers used for vector construction.

Primer name	Primer sequence (from 5′ to 3′)
L5AD5	CGGGTCTCAGGCAGGATGGGCAGTCTGGGCAAC AAAGCACCAGTGG
L3AD5	TAGGTCTCCAAACGGATGAGCGACAGCAAACAA AAAAAAAAGCACCGACTCG
OsARF4-gRNA3(MPK1)-FP	TAGGTCTCCTCTCCTTCAGAGgttttagagctagaa
OsARF4-gRNA3(MPK1)-RP	ATGGTCTCAGAGATGCCTCCTtgcaccagccgggaa
OsARF4-gRNA4(MPK1)-FP	TAGGTCTCCAAGGCTTGTTGCgttttagagctagaa
OsARF6-gRNA4(MPK1)-RP	CGGGTCTCACCTTTTGGAACTtgcaccagccggg
promoter u3	TGGGTACGTTGGAAACCACG
pUBI10	GTTTGTTGGTCGCCGTTAGG
OsARF4(Crispr)-FP	TTTCCCGCGTGAATTCTTCG
OsARF4(Crispr)-RP	CTATGCAAGAATAGCCTAACA
35S:OsARF4-GFP-FP	tacgaattcgagctcggtaccATGCCGCCGGCCGCCAT
35S:OsARF4-GFP-RP	gctcaccatgtcgactctagaGTTATCTGATTTGTTGGGCAT
proOsARF4-GUS-FP	GAGCTCtgcgtgctaaatacagccatac
proOsARF4-GUS-RP	CCATGGctcggatcggggcggcgg

Supplementary Table 2. Primers used for qRT-PCR.

Primer name	Primer sequence (from 5′ to 3′)

OsARF4-RT-FP	CGACCTGAACAGCCAAGAGT
OsARF4-RT-RP	CGGGGAGCTCAAGTGATTGT
OsARF19-RT-FP	GCAGATGAGCTTGGAGGGTT
OsARF19-RT-RP	AGAATGGCCTCGGAGTTCTG
OsIAA1-RT-FP	CACCATCCGGAAGTTCGC
OsIAA1-RT-RP	GTGCTAAGTTGACAGCCTCTG
OsTIR1-RT-FP	CGCTTCCGGATGAAACACCT
OsTIR1-RT-RP	CATTTCCATGTCCTGAAAAAGGCT
OsBZR1-RT-FP	CACGATACCGGAGTGCGAC
OsBZR1-RT-RP	GGGTTGACGAGGTTGTAGGT
D2-RT-FP	AGCTGCCTGGCACTAGGCTCTACAGATCAC
D2-RT-RP	ATGTTGTCGGAGATGAGCTCGTCGGTGAGC
OsACTIN-RT-FP	TCAGCAACTGGGATGATATGGAG
OsACTIN-RT-RP	GCCGTTGTGGTGAATGAGTAAC
